# CircRtn4 Acts as the Sponge of miR-24-3p to Promote Neurite Growth by Regulating CHD5

**DOI:** 10.3389/fnmol.2021.660429

**Published:** 2021-07-07

**Authors:** Yue Qi, Nana Ma, Xiaofan Chen, Yue Wang, Wei Zhang, Jun Wan

**Affiliations:** ^1^Shenzhen Key Laboratory for Neuronal Structural Biology, Biomedical Research Institute, Shenzhen Peking University - The Hong Kong University of Science and Technology Medical Center, Shenzhen, China; ^2^Greater Bay Biomedical Innocenter, Shenzhen Bay Laboratory, Shenzhen, China; ^3^Department of Biology, School of Life Sciences, Southern University of Science and Technology, Shenzhen, China

**Keywords:** circRtn4, miR-24-3p, CHD5, neurite growth, primary cortical neuron, neurodevelopment

## Abstract

Circular RNAs (circRNAs) are covalently closed single-stranded RNA molecules. After derived from precursor mRNA back-splicing, circRNAs play important roles in many biological processes. Recently, it was shown that several circRNAs were enriched in the mammalian brain with unclear functions. The expression of circRtn4 in the mouse brain was increased with the differentiation of primary neurons. In our study, knockdown of circRtn4 inhibited neurite growth, while overexpression of circRtn4 significantly increased neurite length. By dual-luciferase reporter assay and RNA antisense purification assay, circRtn4 was identified as a miRNA sponge for miR-24-3p. Moreover, knockdown of miR-24-3p increased neurite length, while overexpression of miR-24-3p significantly inhibited neurite growth. Furthermore, CHD5 was confirmed to be a downstream target gene of miR-24-3p. And CHD5 silence counteracted the positive effect of circRtn4 overexpression on neurite growth. In conclusion, circRtn4 may act as the sponge for miR-24-3p to promote neurite growth by regulating CHD5.

## Introduction

Billions of specific neurons build the neural circuits, some of which underlie animal behaviors including cognition, learning and feeling. Neurons extend neurites during development, which is a critical step in wiring neural circuits to establish precise neural connections for normal physiological function (Philippidou and Dasen, 2013; Park and Poo, 2013; Duhr et al., 2014; Cave and Sockanathan, 2018). Impaired development of neural circuits was associated with behavioral, cognitive and mental disorders, such as epilepsy, intellectual disability, autism, schizophrenia, etc. (Kaufmann and Moser, 2000; Wong and Guo, 2013; Tang et al., 2014; Levitt and Veenstra-VanderWeele, 2015; MacDonald et al., 2017). Therefore, it is important to elucidate the molecular mechanisms underlying the development of neurons.

Circular RNAs (circRNAs) are a class of non-coding RNAs derived from back-splicing of pre-mRNA. CircRNAs are characterized as covalently closed single-stranded RNA molecules without a 5′ cap and a 3′ polyadenylated tail (Wilusz, 2018). Since the first circRNA was identified over 40 years ago, it had been dismissed as noise without any biological function for a long time. Numerous recent reports have shown that circRNAs play vital roles in diverse pathophysiological conditions, including neurodegenerative diseases (Kumar et al., 2017), cancer (Wang et al., 2018; Lu et al., 2019; Zhang et al., 2019) and other diseases (You et al., 2015; Garikipati et al., 2019). Moreover, circRNA enriched in the nucleus could interact with RNA polymerase II holocomplex to regulate transcription (Li et al., 2015). While in the cytoplasm, circRNAs exert their functions though sponging microRNAs (miRNAs) (Hansen et al., 2013), binding RNA binding protein (Garikipati et al., 2019) and undergoing protein translation (Legnini et al., 2017).

MiRNAs, with a length of about 22 nucleotides, are a class of small non-coding single-stranded RNA molecules highly conserved across species. In addition, most of miRNAs negatively regulate the expression of protein-coding genes by binding to their 3′-untranslated region (3′ UTR) (Bartel, 2004). The crucial roles of miRNAs in neural development indicated their potential applications in the therapeutic methods of neurodevelopmental related diseases (Fineberg et al., 2009; Im and Kenny, 2012; Mahmoudi and Cairns, 2017; McNeill et al., 2020).

Mmu_circ_0000250 (also called as circRtn4) arises from the head-to-tail splicing of exon 2 and exon 3 of Rtn4 gene, which is 2,418 base pairs in length. The expression of circRtn4 is high in mouse brain, which increases with neuronal differentiation (Rybak-Wolf et al., 2015). However, its role in neurodevelopment is not well elucidated. In this study, we found that circRtn4 may promote neurite growth by sponging miR-24-3p to regulate CHD5.

## Materials and Methods

### Animals

The C57BL/6J mice were purchased from the Medical Experimental Animal Center of Guangdong Province. All mice were housed in separate cages with free access to food and water. The rooms were maintained with 12-h light/dark cycle at constant temperature and humidity. All procedures of animal experiments were conformed to animal use protocols and approved by the Committee for the Ethics of Animal Experiments, Shenzhen-Peking University-The Hong Kong University of Science and Technology Medical Center (SPHMC; protocol number 2011-004).

### Cell Cultures and Transfection

Mouse neuroblastoma cell line Neuro2A cells (N2a) were maintained in the growth medium composed of Minimum Essential Medium (MEM) with 10% Fetal Bovine Serum (FBS, Gibco, United States). The differentiation of N2a cells was induced by the differentiation medium composed of Dulbecco’s Modified Eagle’s Medium (DMEM), 0.5% FBS and 10 μM retinoic acid (RA) (Sigma, United States). HEK293T cells were cultured in DMEM containing 10% FBS. Primary cortical neurons were isolated from E16.5–18.5 C57BL/6 mice embryos and seeded on poly-L-lysine (Sigma)—coated Petri dishes. Primary cortical neurons were seeded in DMEM with 10% FBS and 1% Penicillin/Streptomycin for the first 4 h, then in Neurobasal Medium containing 2% B27 (Life Technologies, United States) and 0.5 mM L-glutamine for further culture. All cells were cultured at 37°C in an incubator with 5% CO_2_. For siRNA or miRNA transfection of each well, 100 nM siRNA (RiboBio, China), 100 nM miRNA mimic (Ribobio, China) or 100 nM miRNA inhibitor (Ribobio, China) was added with RNA iMAX Reagent (Sigma, United States). For plasmid transfection, 750 ng/ml plasmids were added with ViaFect^TM^ Transfection Reagent (Promega, United States) according to the manufacturer’s guidelines. After 24 h of transfection, N2a cells were induced differentiation by changing of differentiation medium and harvested 48 h later. Primary cortical neurons were harvested after 72 h of transfection. The siRNAs used in this study can be found in Supplementary Table 2.

### Quantitative Real-Time PCR (qRT-PCR)

Total mRNA was extracted from cells with RNA-easy Isolation Reagent (Vazyme, China) according to the manufacturer’s instructions. Cytoplasmic and nuclear RNA were isolated by Nuclear and Cytoplasmic Protein Extraction Kit (BD, China) following the manufacturer’s protocol. For RNase R treatment, total RNA was incubated for 10 min at 37°C with or without 3.5 U RNase R (Lucigen, United States). Reverse transcription was performed with the Goscript RT system (Promega, United States), and quantitative PCR was performed with SYBR Green Realtime-PCR Master Mix (BIO-RAD, United States). The divergent primers for circRtn4 were designed to amplify across the backsplicing junction, while the Rtn4 primers were designed to amplify the sequence of linear-Rtn4 mRNA not present in circRtn4. Glyceraldehyde-3-phosphate dehydrogenase (GAPDH) was used as a loading control for circRtn4 and other mRNA, while the expression of miRNA was normalized to U6 in each sample. The relative changes in gene expression were calculated by the relative quantification method (2^−△△Ct^). The primers used in this study were listed in Supplementary Table 1.

### Fluorescence *in situ* Hybridization (FISH)

Alexa Fluor 555-labeled probes of circRtn4, U6 and 18S are purchased from Advanced Cell Diagnostics (United States). The probe signals were detected with Ribo^TM^ Fluorescent *in situ* Hybridization kit (Ribobio, China) following the manufacturer’s instructions. The probes of U6 and 18S were used as control. The results were observed with a Zeiss LSM 710 confocal microscope with a 40 × objective.

### Plasmid Construction

The sequence of circRtn4 was amplified from mouse cDNA, and then its binding sites of miR-24-3p were mutated. Wild type or mutant circRtn4 sequence were inserted into psiCHECK^TM^-2 Vector (Promega, United States) or pcDNA 3.1 vector using ClonExpress^®^ MultiS One Step Cloning Kit (Vazyme, China) according to the manufacturer’s instructions. The sequences of all constructs were tested by DNA sequencing (IGE Biotech, China).

### Fluorescence Immunostaining

Cells were fixed in 4% paraformaldehyde (PFA) for 15 min at 4°C, then permeabilized and blocked in PBS containing 1% BSA, 4% goat serum, and 0.4% Triton X-100 for 1 h at room temperature. Subsequently, cells were incubated with primary antibody (anti-β-tubulin III, 1:1000, Sigma, mouse monoclonal antibody) overnight at 4°C, followed with secondary antibody (anti-mouse, CY3, 1:400, Jackson Immuno Research) for 2 h at room temperature. Images of cells were captured by a Zeiss LSM 710 confocal microscope with a 20 × objective. Images were analyzed with Zen 2012 (Zeiss) and Image-Pro Plus.

### Dual Luciferase Reporter Assay

HEK-293T cells were seeded in 12-well plates and cotransfected with 200 ng psiCHECK^TM^-2-circRtn4-WT, psiCHECK^TM^-2-circRtn4-MUT or psiCHECK^TM^-2-control and 200 nM miRNA mimics or miRNA negative control using ViaFect^TM^ Transfection Reagent (Promega, United States). At 30 h after transfection, cells were harvested and luciferase activity was detected with the dual luciferase reporter assay system (Promega, United States). Renilla luciferase activity was measured, and the values were normalized to firefly luciferase activity.

### RNA Antisense Purification (RAP) Assay

The biotin-labeled probes of circRtn4 and control were designed by BersinBio (China). More than 4 × 10^7^ N2a cells were harvested and RAP assay was performed using the RIP kit (BersinBio, China) according to the manufacturer’s instructions. After hybridized with probes, the circPCMTD1 and control complex were captured. And then, the miRNAs in these RNA complexes were purified and detected by qRT-PCR. The probes used in this study were listed in Supplementary Table 3.

### Statistical Analysis

Data was analyzed by SPSS 19.0 and GraphPad Prism 7.0. Comparisons among multiple groups were calculated using one-way ANOVA, followed by LSD’s *post hoc* analysis. Comparisons between two groups were performed with Student’s unpaired *t*-test. The data presented as mean ± SD. *P* < 0.05 was considered statistically significant.

## Results

### CircRtn4 Characterization and Structure Identification

**FIGURE 1:**
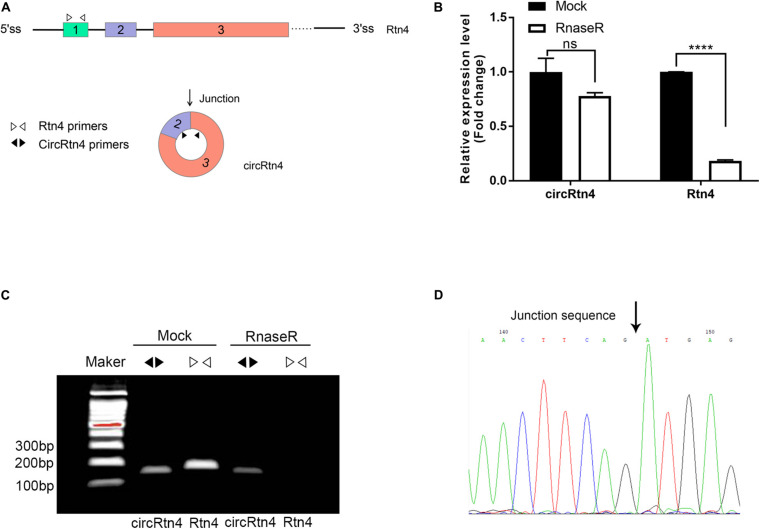
CircRtn4 characterization and structure identification. **(A)** Schematic diagram of circRtn4 primers and Rtn4 primers. **(B)** qRT-PCR was performed to measure the expression levels of circRtn4 and linear-Rtn4 mRNA treated with or without RNase R. The results were shown as the mean ± SD (****p < 0.0001, *n* = 3). **(C)** The existence of circRtn4 in N2a cells was confirmed by RT-PCR. **(D)** The head-to-tail splicing sites of circRtn4 was validated by Sanger sequencing.

In this study, several methods were applied to avoid the head-to-tail splicing products from trans-splicing or genomic rearrangements. First, we designed circRtn4 primers and Rtn4 primers. CircRtn4 primers were divergent primers for specific back-splicing junction. Rtn4 primers were designed in exon 1 of Rtn4 mRNA (Figure 1A). Subsequently, we performed qRT-PCR assay and found that circRtn4, rather than linear-Rtn4, was resistant to RNase R digestion (Figure 1B). Furthermore, the resistance of circRtn4 to RNase R was also confirmed by agarose gel electrophoresis (Figure 1C). The circRtn4 and Rtn4 fragments longth are 157 and 189 bp, respectively. Moreover, the head-to-tail splicing in the RT-PCR product of circRtn4 was confirmed by Sanger sequencing, which is consistent with the data in the CircBase database (Figure 1D).

### CircRtn4 Promotes Neurite Growth

**FIGURE 2:**
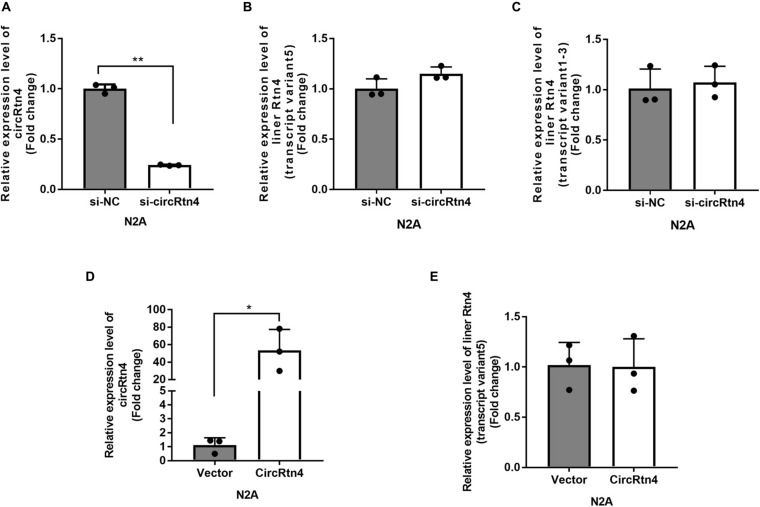
The efficiency of circRtn4 knockdown and overexpression. **(A)** The knockdown efficiency of circRtn4 was determined by qRT-PCR. **(B,C)** After transfection of circRtn4 siRNA, the expression levels of Rtn4 variants were detected by qRT-PCR. The results were shown as the mean ± SD (*n* = 3). **(D)** The overexpression efficiency of circRtn4 was determined by qRT-PCR. **(E)** After transfection of overexpressing vector, the expression levels of Rtn4 variant 5 were detected by qRT-PCR. The results were shown as the mean ± SD (*p < 0.05, and **p < 0.01 *n* = 3).

It was reported that the expression level of circRtn4 was significantly increasing during the neuronal differentiation (Rybak-Wolf et al., 2015). Similarly, we found that the expression level of circRtn4 increased significantly after the differentiation of N2a cells and primary cortical neurons (Supplementary Figures 1A,B), which indicates the potential role of circRtn4 in this process. The siRNA-mediated knockdown of circRtn4 was efficient in N2a cells, while the expression of Rtn4 linear mRNA had no significant change (Figures 2A–C). Then, the plasmid for circRtn4 overexpression was derived from pcDNA3.1, which expressed green fluorescent protein (GFP). The plasmids transiently transfected into N2a cells increased circRtn4 levels (Figure 2D), instead of linear-Rtn4 mRNA levels (Figure 2E).

**FIGURE 3:**
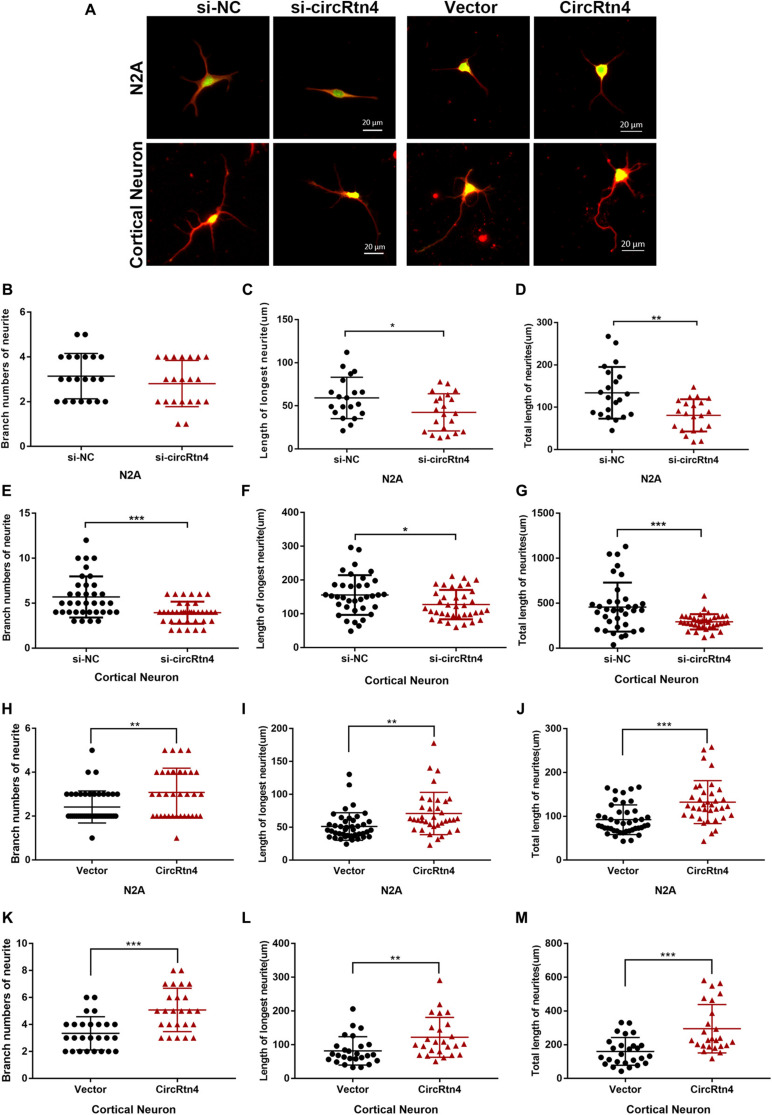
CircRtn4 promotes neurite growth. **(A)** Following circRtn4 knockdown or overexpression, N2a cells and primary cortical neurons were fixed and immunostained with anti-β-tubulin III antibody and captured by a Zeiss LSM 710 confocal microscope with a 20 × objective (Red, β-tubulin III; Green, GFP). N2a cells **(B–D)** and cortical neurons **(E–G)** were cotransfected with siRNAs (100 nM) and PEGFP vector (250 ng/ml). N2a cells **(H–J)** and cortical neurons **(K–M)** were transfected with overexpression vector of circRtn4 (750 ng/ml). The branch numbers of neurite, the length of longest neurite and total length of neurites were quantified by Image-Pro Plus software. The results were shown as the mean ± SD (*p < 0.05, **p < 0.01, and ***p < 0.001).

After N2a cells were transfected with circRtn4 siRNA, their length of longest neurite and total length of neurites were significantly decreased, while their branch numbers showed no change (Figures 3A–D). Knockdown of circRtn4 in cortical neurons showed similar effects on their longest neurite length and total neurites length, while their branch numbers were reduced (Figures 3A,E–G). Otherwise, both N2a cells and cortical neurons overexpressing circRtn4 exhibited increased branch numbers, increased length of the longest neurite and increased total length of neurites (Figures 3A,H–M).

### CircRtn4 Functions as a Sponge of miR-24-3p

**FIGURE 4:**
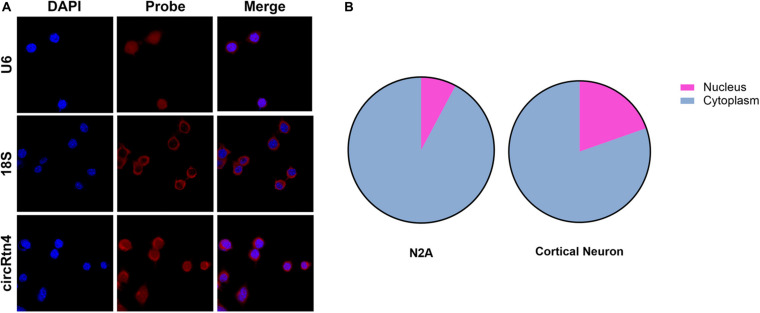
The localization of circRtn4 in N2a cells. **(A)** RNA FISH showed the localization of circRtn4 in N2a cells. **(B)** After nuclear and cytoplasmic isolation in N2a cells and primary cortical neurons, the expression levels of circRtn4 were examined by qRT-PCR.

**FIGURE 5:**
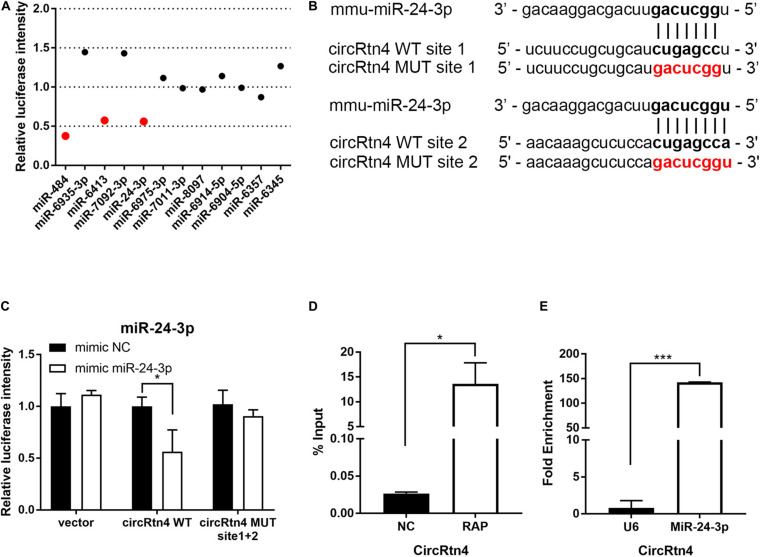
CircRtn4 functions as a sponge of miR-24-3p. **(A)** 12 miRNAs candidates were screened by Dual-luciferase reporter assay. **(B)** The wild-type (WT) and mutated (MUT) circRtn4 binding sites with miR-24-3p were shown. **(C)** HEK-293T cells were cotransfected with psiCHECK^TM^-2-circRtn4-WT, psiCHECK^TM^-2-circRtn4-MUT or psiCHECK^TM^-2-control and miRNA mimics or miRNA negative control. Luciferase activity was detected with luciferase reporter assays. **(D)** The enrichment efficiency of circRtn4 probe was confirmed by qRT-PCR. **(E)** The enrichment of miR-24-3p in the circRtn4 RAP assay was detected by qRT-PCR. The results were shown as the mean ± SD (*p < 0.05, and ***p < 0.001 *n* = 3).

To gain insight into the molecular mechanism of circRtn4 promoting neurite growth, fluorescent *in situ* hybridization (FISH) experiments were conducted. CircRtn4 was abundant in the cytoplasmic N2a cells (Figure 4A), which was also confirmed by nuclear and cytoplasmic isolation in N2a cells and primary cortical neurons (Figure 4B). It indicated circRtn4 may function as a miRNA sponge. Then, dual-luciferase reporter assay was performed to screen 12 miRNAs candidates predicted by miRDB. It was found miR-484, miR-6413 and miR-24-3p can interact with circRtn4 directly (Figure 5A). Among them, only miR-24-3p was associated with neurite growth in previously reports (Kang et al., 2019). Transfection of miR-24-3p mimic effectively weakened the activity of luciferase reporter containing wild type circRtn4. Two binding sites in circRtn4 sequence were mutated as shown in Figure 5B. However, miR-24-3p did not affect the activity of luciferase reporter containing mut-circRtn4 (Figure 5C). Furthermore, a 142.5-fold enriched miR-24-3p was captured with the biotin-labeled circRtn4 in RAP assay (Figures 5D,E), which revealed that circRtn4 could act as a sponge of miR-24-3p.

### CircRtn4 Promotes Neurite Growth via miR-24-3p

**FIGURE 6:**
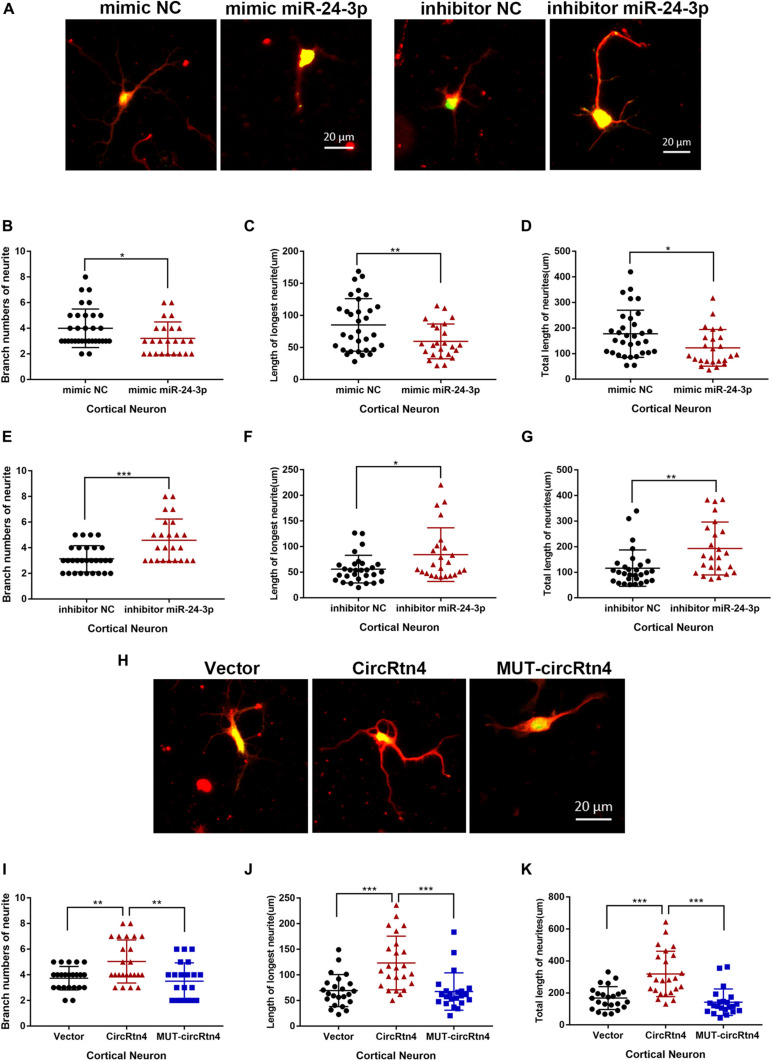
CircRtn4 promotes neurite growth via miR-24-3p. **(A)** After transfected with miR-24-3p inhibitor or miR-24-3p mimic, primary cortical neurons were fixed and immunostained with anti-β-tubulin III antibody and captured by a Zeiss LSM 710 confocal microscope with a 20 × objective (Red, β-tubulin III; Green, GFP). Primary cortical neurons were cotransfected with 100 nM miR-24-3p mimic **(B–D)** or inhibitor **(E–G)** and PEGFP vector (250 ng/ml). The branch numbers of neurite, the length of longest neurite and total length of neurites were quantified by Image-Pro Plus software. The results were shown as the mean ± SD (*p < 0.05, **p < 0.01, and ***p < 0.001). **(H)** After transfected with circRtn4 or MUT-circRtn4 vector, primary cortical neurons were fixed and immunostained with anti-β-tubulin III antibody and captured by a Zeiss LSM 710 confocal microscope with a 20 × objective (Red, β-tubulin III; Green, GFP). **(I–K)** Primary cortical neurons were transfected with overexpression vector of circRtn4 or MUT-circRtn4 (750 ng/ml). The branch numbers of neurite, the length of longest neurite and total length of neurites were quantified by Image-Pro Plus software. The results were shown as the mean ± SD (*p < 0.05, **p < 0.01, and ***p < 0.001).

Corresponding to the increased expression of circRtn4, the expression level of miR-24-3p decreased observably with the differentiation of N2a cells and primary cortical neurons (Supplementary Figures 1C,D). To explore the function of miR-24-3p in neurite growth, miR-24-3p mimic or miR-24-3p inhibitor was transfected into N2a cells and cortical neurons, of which the efficiency was verified by qRT-PCR (Supplementary Figures 2A,B). Cortical neurons overexpressing miR-24-3p displayed obviously reduced growth and branching, whereas the induction of miR-24-3p inhibitor significantly increased branch number, the longest neurite length and total neurite length (Figures 6A–G). Similarly, the negative effect of miR-24-3p on neurite growth was also confirmed in N2a cells (Supplementary Figures 3A–G). To further identify whether circRtn4 improves neurite growth as a sponge of miR-24-3p, circRtn4 mutant form Mut-circRtn4 were overexpressed in cortical neurons and N2a cells. The promoting effect of circRtn4 on neurite growth did not exist in neither cortical neurons nor N2a cells overexpressing Mut-circRtn4 (Figures 6H–K and Supplementary Figures 3H–K), which indicated that circRtn4 promotes neurite growth at least partly by miR-24-3p.

### CHD5 Is a Target Gene of miR-24-3p Regulated by circRtn4

**FIGURE 7:**
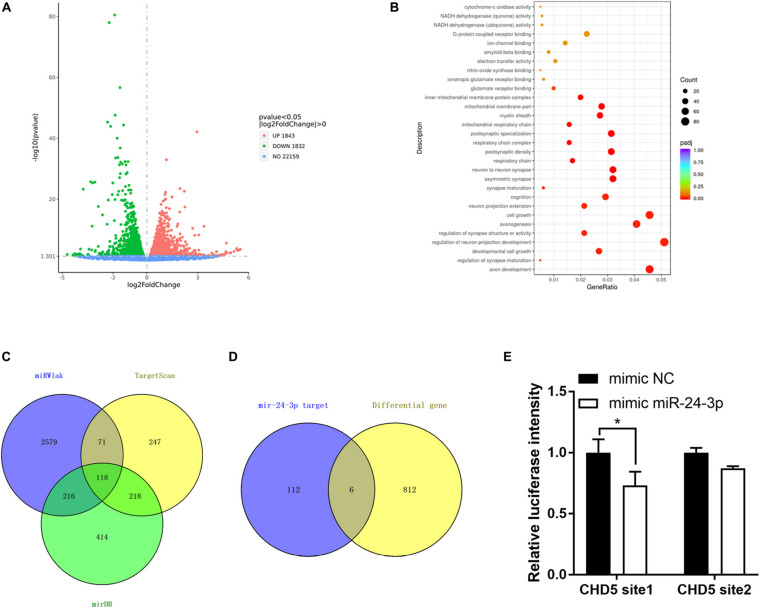
CHD5 is the target gene of miR-24-3p. **(A)** The variation of genes expression in N2a cells transfected si-circRtn4 were revealed in the scatter plot. **(B)** Gene Ontology analyses of downregulated genes. **(C)** The Venn diagram showed the potential target genes predicted by databases. **(D)** The Venn diagram showed the same genes between genes predicted by databases and differential genes in transcriptome sequencing. **(E)** HEK-293T cells were cotransfected with psiCHECK^TM^-2-CHD5 site1, psiCHECK^TM^-2-CHD5 site2 or psiCHECK^TM^-2-control and miRNA mimics or miRNA negative control. Luciferase activity was detected with luciferase reporter assays. The results were shown as the mean ± SD (*p < 0.05).

To investigate the potential involved mRNAs, transcriptome RNA sequencing was employed to identify differential genes after circRtn4 knockdown in N2a cells. The raw data can be found at the NCBI GEO with accession number GSE165253. There were 1,843 upregulated genes and 1,832 downregulated genes in N2a cells when circRtn4 was silenced (Figure 7A). Most downregulated genes were involved in neural development (Figure 7B). We predicted the target genes of miR-24-3p by cross analyzing three prediction databases (miRWalk, TargetScan, mirDB) and 118 genes were predicted in all three databases (Figure 7C). Then we compared 818 downregulated genes with | fold change| > 0.5 (*P* < 0.05) and the 118 mRNAs of database projections using Venn diagram. There were 6 genes in the overlapped spaces in the Venn diagram (Figure 7D). We performed dual-luciferase reporter assay to screen 6 genes. And we found the activity of luciferase reporter containing CHD5 was effectively reduced by miR-24-3p mimic (Figure 7E), which suggested that CHD5 is the target gene of miR-24-3p.

### CHD5 Reversed the Neurite Growth Promotion Mediated by circRtn4

**FIGURE 8:**
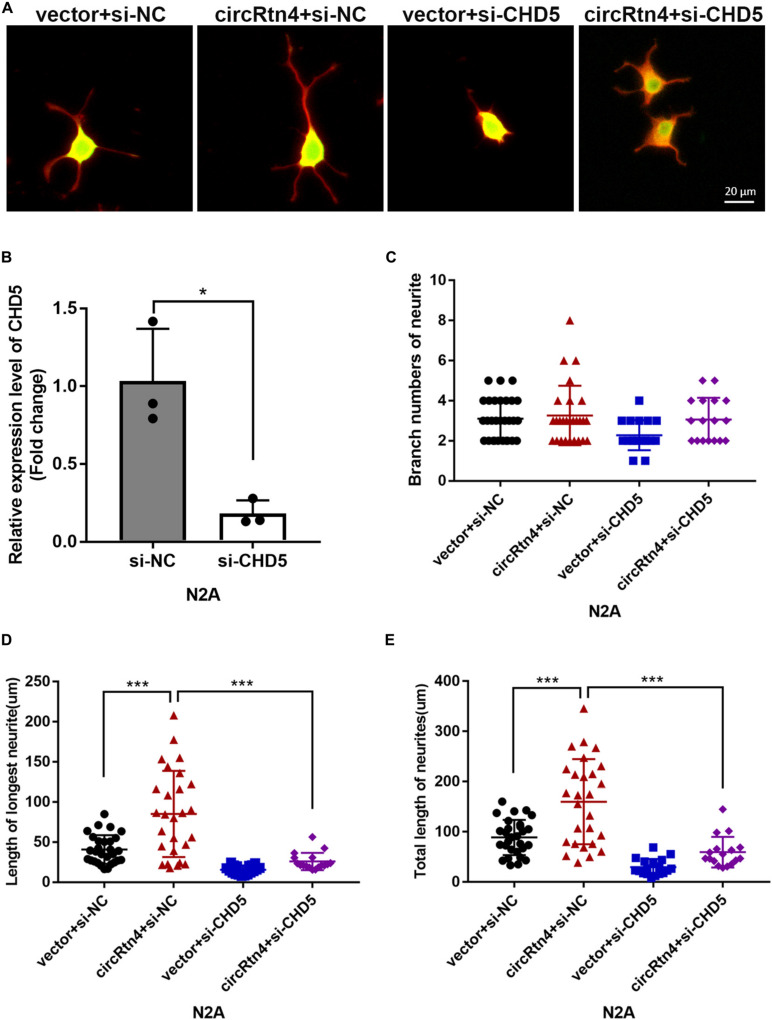
CHD5 reversed the neurite growth promotion mediated by circRtn4. **(A)** After transfected with vector + si-NC, circRtn4 + si-NC, vector + si-CHD5 and circRtn4 + si-CHD5, N2a cells were fixed and immunostained with anti-β-tubulin III antibody and captured by a Zeiss LSM 710 confocal microscope with a 20 × objective (Red, β-tubulin III; Green, GFP). N2a were cotransfected with 100 nM siRNA and 500 ng/ml pcDNA3.1 vector. **(B)** The knockdown efficiency of CHD5 was determined by qRT-PCR (*n* = 3). **(C–E)** The branch numbers of neurite, the length of longest neurite and total length of neurites were quantified by Image-Pro Plus software. The results were shown as the mean ± SD (*p < 0.05, and ***p < 0.001).

Similar to circRtn4, the differentiation of N2a cells and primary cortical neurons increased the expression level of CHD5 (Supplementary Figures 1E,F). We further examined whether CHD5 is downstream gene regulated by circRtn4 to promote neurite growth. N2a cells were transfected with vector + si-NC, circRtn4 + si-NC, vector + si-CHD5 and circRtn4 + si-CHD5. The knockdown efficiency of CHD5 was determined by qRT-PCR (Figure 8B). As previously observed, circRtn4 overexpression effectively promote neurite growth. However, CHD5 silence reversed the promoting effect of circRtn4 (Figures 8A,C–E).

## Discussion

CircRNAs abundant in mammalian brain, especially in neurons, may play important roles in the nervous system (You et al., 2015; Rybak-Wolf et al., 2015; Chen and Schuman, 2016; Xu et al., 2020). For instance, cirTLK1 aggravates neuronal injury and neurological deficits after ischemic stroke (Wu et al., 2019). Circ_0000950 promotes neuron apoptosis and suppresses neurite outgrowth in Alzheimer’s disease (Yang et al., 2019). CircPTK2 regulates oxygen-glucose deprivation-activated microglia-induced hippocampal neuronal apoptosis (Wang et al., 2019). Moreover, circDLGAP4 exerts neuroprotective effects (Feng et al., 2020).

It was reported that significantly increased expression of circRtn4 is associated with the differentiation of primary neurons (Rybak-Wolf et al., 2015). In our study, we explored the role of circRtn4 in neurite growth and found it promote neurite branching and growth. Furthermore, circRtn4 mainly located in the cytoplasm, where circRNAs often function as miRNA sponges (Wilusz, 2018). As expected, circRtn4 interacted directly with miR-24-3p.

In recent years, miR-24-3p has been reported to be involved in the pathogenesis of cardiovascular disease (Li et al., 2020; Qiao et al., 2020). More studies have focused on its roles in cell apoptosis, proliferation, migration and invasion in various cancers, including glioma (Xu et al., 2013), lung cancer (Yan et al., 2018), bladder cancer (Yu et al., 2017) and hepatocellular carcinoma (Fan et al., 2018). In our study, circRtn4 was considered as the sponge of miR-24-3p, which promotes the differentiation of neural cells. Moreover, dysregulation of miR-24-3p significantly affected the neurite branching and growth, which is consistent with its role in inhibiting neuronal differentiation (Kang et al., 2019). In this report, miR-24-3p was found to modulate neurite outgrowth by targeting HPCA in SH-SY5Y cells (Kang et al., 2019). However, the binding site of miR-24-3p on HPCA was poorly conserved between human and mouse. Therefore, the target genes of miR-24-3p in the mouse neurodevelopment need to be further explored.

Chromodomain helicase DNA binding protein 5 (CHD5) is well known as a tumor suppressor gene involved in multiple tumors (Fujita et al., 2008; Wong et al., 2011; Wu et al., 2012; Zhao et al., 2012; Wang et al., 2013). Evidence emerged that miR-24-3p targeted CHD5 to promote cells proliferation and regulate chemosensitivity in head and neck squamous cell carcinoma (Sun et al., 2016). Similarly, we proved that CHD5 is a target gene of miR-24-3p. In addition to its antitumor role, CHD5 was reported to be critical to neuronal differentiation (Egan et al., 2013). In our study, knockdown of Chd5 blocked neuronal differentiation, which was enough to reverse the promoting role of circRtn4 overexpression in neuronal differentiation.

Collectively, our findings showed that circRtn4 regulates neurite growth by sponging miR-24-3p to enhance CHD5, which supplied new insights into the molecular mechanisms of neurodevelopmental regulation. Moreover, circRtn4 may be a promising biomarker and a potential therapeutic target.

## Data Availability Statement

The datasets presented in this study can be found at the NCBI GEO with accession number GSE165253.

## Ethics Statement

The animal study was reviewed and approved by the Shenzhen-Peking University-The Hong Kong University of Science and Technology Medical Center.

## Author Contributions

YQ carried out the molecular and cellular studies, participated in the animal experiments, and drafted the manuscript. NM, XC, and YW participated in the circRtn4 structure identification and performed FISH and nuclear and cytoplasmic isolation assays revised the manuscripts. WZ and JW conceived of the study and helped to draft the manuscripts. All authors have read and approved the final manuscript.

## Conflict of Interest

The authors declare that the research was conducted in the absence of any commercial or financial relationships that could be construed as a potential conflict of interest.

## Supplementary Material

Supplementary Figure 1 Expression levels of circRtn4, miR-24-3p and CHD5 during neuronal differentiation. The expression levels of circRtn4 **(A,B)**, miR-24-3p **(C,D)** and CHD5 **(E,F)** in N2a cells **(A,C,E)** and primary cortical neurons **(B,D,F)** at different differentiation time points were determined by qRT-PCR (*n* = 3, ^∗^*p* < 0.05, ^∗∗^*p* < 0.01, and ^∗∗∗^*p* < 0.001).

Supplementary Figure 2 The efficiency of miR-24-3p knockdown and overexpression. **(A)** The overexpression efficiency of miR-24-3p was determined by qRT-PCR. **(B)** After transfection of miR-24-3p inhibitor, the expression levels of miR-24-3p were detected by qRT-PCR. The results were shown as the mean ± SD (^∗^*p* < 0.05, and ^∗∗^*p* < 0.01, *n* = 3).

Supplementary Figure 3 CircRtn4 promotes neurite growth via miR-24-3p. **(A)** After transfected with miR-24-3p inhibitor or miR-24-3p mimic, N2a cells were fixed and immunostained with anti-β-tubulin III antibody and captured by a Zeiss LSM 710 confocal microscope with a 20 × objective (Red, β-tubulin III; Green, GFP). N2a cells were cotransfected with 100 nM miR-24-3p mimic **(B–D)** or inhibitor **(E–G)** and PEGFP vector (250 ng/ml). The branch numbers of neurite, the length of longest neurite and total length of neurites were quantified by Image-Pro Plus software. The results were shown as the mean ± SD (^∗^*p* < 0.05, ^∗∗^*p* < 0.01, and ^∗∗∗^*p* < 0.001). **(H)** After transfected with circRtn4 or MUT-circRtn4 vector, N2a cells were fixed and immunostained with anti-β-tubulin III antibody and captured by a Zeiss LSM 710 confocal microscope with a 20 × objective (Red, β-tubulin III; Green, GFP). **(I–K)** N2a cells were transfected with overexpression vector of circRtn4 or MUT-circRtn4 (750 ng/ml). The branch numbers of neurite, the length of longest neurite and total length of neurites were quantified by Image-Pro Plus software. The results were shown as the mean ± SD (^∗^*p* < 0.05, ^∗∗^*p* < 0.01, and ^∗∗∗^*p* < 0.001).

## Abbreviations

CircRNAs: circular RNAs
MiR: microRNA
CHD5: chromodomain helicase DNA binding protein 5.
