# Supplementary figures and images for: CircRtn4 Acts as the Sponge of miR-24-3p to Promote Neurite Growth by Regulating CHD5
Source: Front Mol Neurosci. 2021 Jul 7;14:660429. doi: 10.3389/fnmol.2021.660429 (PMC8294096; doi:10.3389/fnmol.2021.660429)

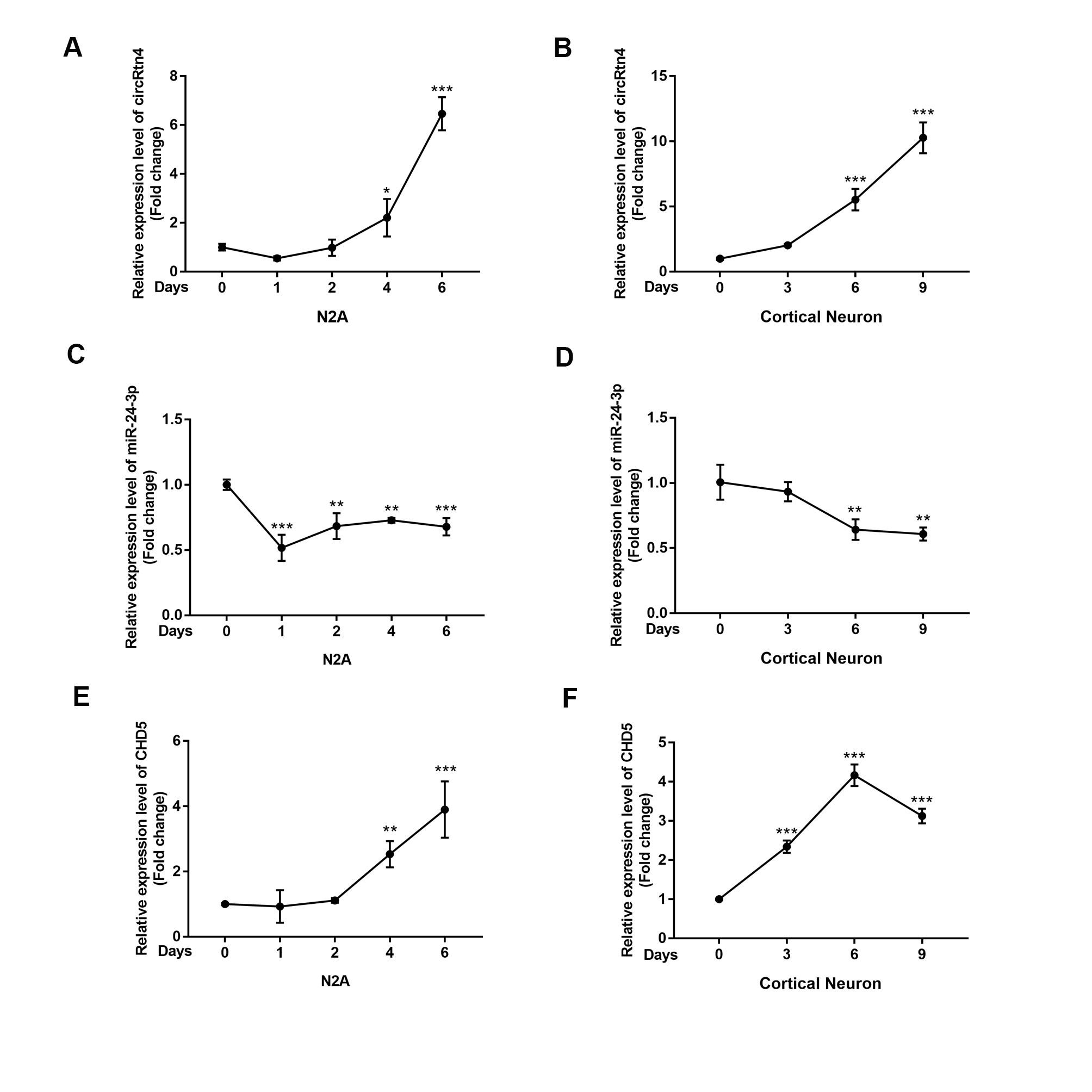

Supplement: Supplementary Figure 1 — Expression levels of circRtn4, miR-24-3p and CHD5 during neuronal differentiation. The expression levels of circRtn4 (A,B), miR-24-3p (C,D) and CHD5 (E,F) in N2a cells (A,C,E) and primary cortical neurons (B,D,F) at different differentiation time points were determined by qRT-PCR (n = 3, ∗p < 0.05, ∗∗p < 0.01, and ∗∗∗p < 0.001). [file Image_1.TIF]

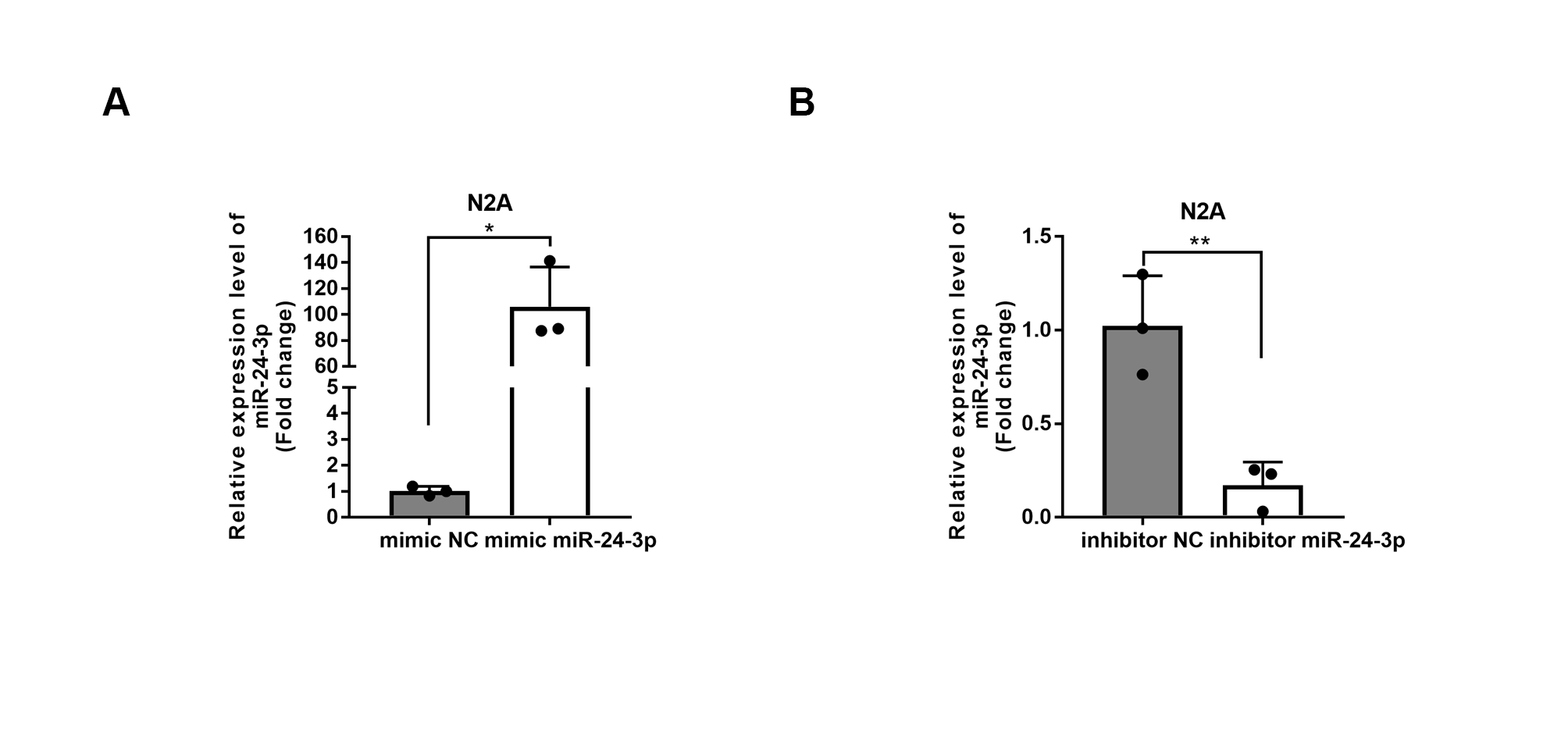

Supplement: Supplementary Figure 2 — The efficiency of miR-24-3p knockdown and overexpression. (A) The overexpression efficiency of miR-24-3p was determined by qRT-PCR. (B) After transfection of miR-24-3p inhibitor, the expression levels of miR-24-3p were detected by qRT-PCR. The results were shown as the mean ± SD (∗p < 0.05, and ∗∗p < 0.01, n = 3). [file Image_2.TIF]

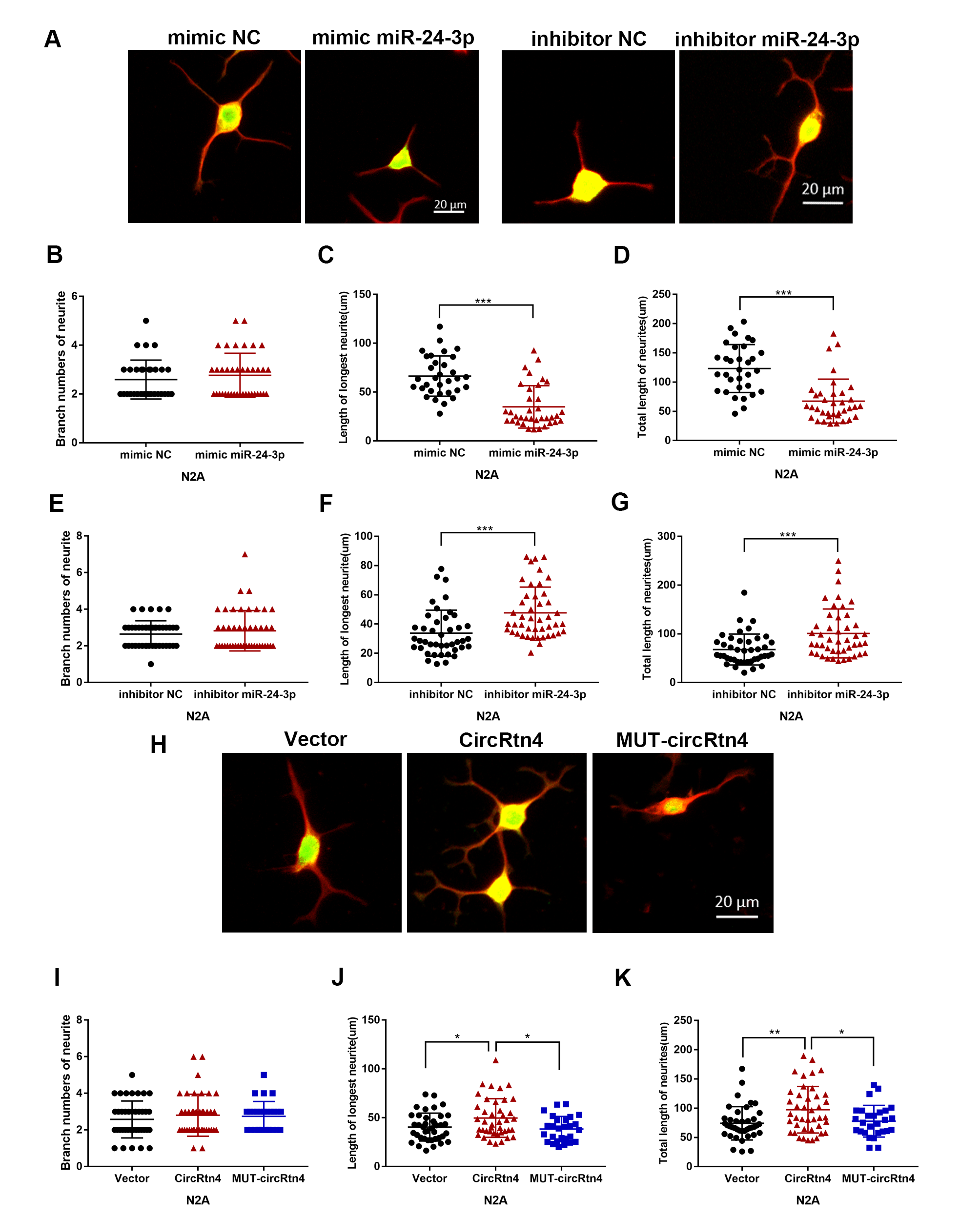

Supplement: Supplementary Figure 3 — CircRtn4 promotes neurite growth via miR-24-3p. (A) After transfected with miR-24-3p inhibitor or miR-24-3p mimic, N2a cells were fixed and immunostained with anti-β-tubulin III antibody and captured by a Zeiss LSM 710 confocal microscope with a 20 × objective (Red, β-tubulin III; Green, GFP). N2a cells were cotransfected with 100 nM miR-24-3p mimic (B–D) or inhibitor (E–G) and PEGFP vector (250 ng/ml). The branch numbers of neurite, the length of longest neurite and total length of neurites were quantified by Image-Pro Plus software. The results were shown as the mean ± SD (∗p < 0.05, ∗∗p < 0.01, and ∗∗∗p < 0.001). (H) After transfected with circRtn4 or MUT-circRtn4 vector, N2a cells were fixed and immunostained with anti-β-tubulin III antibody and captured by a Zeiss LSM 710 confocal microscope with a 20 × objective (Red, β-tubulin III; Green, GFP). (I–K) N2a cells were transfected with overexpression vector of circRtn4 or MUT-circRtn4 (750 ng/ml). The branch numbers of neurite, the length of longest neurite and total length of neurites were quantified by Image-Pro Plus software. The results were shown as the mean ± SD (∗p < 0.05, ∗∗p < 0.01, and ∗∗∗p < 0.001). [file Image_3.TIF]
