# Supplementary material for: CircRtn4 Acts as the Sponge of miR-24-3p to Promote Neurite Growth by Regulating CHD5
Source: Front Mol Neurosci. 2021 Jul 7;14:660429. doi: 10.3389/fnmol.2021.660429 (PMC8294096; doi:10.3389/fnmol.2021.660429)
Supplement: Supplementary file 4 [file Table_1.DOCX]

**Supplementary Tables**

**Table S1 Primers used for RT-qPCR.**

| **Primer name** | **Sequence（5’-3’）** |
| --- | --- |
| CircRtn4-F | AGATCCCTGACAGCTGTATTGT |
| CircRtn4-R | CTTGACCAGACGAAACAGTGT |
| Rtn4 (transcript variant 5)-F | ACCCTAGGGCTTTGTCTACG |
| Rtn4 (transcript variant 5)-R | TCCTAGCTGCTGATAGGCGA |
| Rtn4 (transcript variant 1-3)-F | GAGGCAGGAGGAGAAGTCTT |
| Rtn4 (transcript variant 1-3)-R | AACTGGTACTTGAAAGCGGG |
| mouse-GAPDH-F | AACTTTGGCATTGTGGAAGG |
| mouse-GAPDH-R | ATGCAGGGATGATGTTCT |
| U6-F | GCTTCGGCAGCACATATACTAAAT |
| U6-R | CGCTTCACGAATTTGCGTCTCAT |
| miR-24-3p-qPCR-F | GCGTGGCTCAGTTCAGCAG |
| miR-24-3p-qPCR-R | AGTGCAGGGTCCGAGGTATT |
| miR-24-3p-RT | GTCGTATCCAGTGCAGGGTCCGAGGTATTCGCACTGGATACGACCTGTTC |
| CHD5-F | AGCTCCCTGGTCAAGTCTTC |
| CHD5-R | CAGGAGCGTTGAATCTGTCG |
|  |  |

**Table S2 The siRNAs used in this study.**

| **Name** | **Probe sequence（5’-3’）** |
| --- | --- |
| si-circRtn4 | CTTCAGATGAGACCCTTTT |
| si-CHD5 | GTATCGCAACTACCAAAGA |

**Table S3 The probes used in the RAP assay**

| **Name** | **Probe sequence（5’-3’）** |
| --- | --- |
| circRtn4 | ATGCAGCAGGAAGAGCAAAAAGGGTCTCATCTGAAGTTTTATTCAGCTCTGCTGACAATA |
| Lac Z | CAAACGGCGGATTGACCGTAATGGGATAGGTCACGTTGGTGTAGATGGGCGCATCGTAAC |
| Lac Z | CACCACATACAGGCCGTAGCGGTCGCACAGCGTGTACCACAGCGGATGGTTCGGATAATG |
| Lac Z | CCAATCCGCGCCGGATGCGGTGTATCGCTCGCCACTTCAACATCAACGGTAATCGCCATT |
